# Uric Acid Induces Endothelial Dysfunction by Activating the HMGB1/RAGE Signaling Pathway

**DOI:** 10.1155/2017/4391920

**Published:** 2017-01-01

**Authors:** Wei Cai, Xi-Mei Duan, Ying Liu, Jiao Yu, Yun-Liang Tang, Ze-Lin Liu, Shan Jiang, Chun-Ping Zhang, Jian-Ying Liu, Ji-Xiong Xu

**Affiliations:** ^1^Department of Endocrinology and Metabolism, First Affiliated Hospital of Nanchang University, Nanchang, Jiangxi 330006, China; ^2^Department of Medical Genetics and Cell Biology, Medical College of Nanchang University, Nanchang, Jiangxi 330006, China; ^3^Department of Endocrinology and Metabolism, People's Hospital of Shangrao City, Shangrao, Jiangxi 334600, China; ^4^Department of Endocrinology and Metabolism, Fourth Affiliated Hospital of Nanchang University, Nanchang, Jiangxi 330003, China

## Abstract

Uric acid (UA) is a risk factor for endothelial dysfunction, a process in which inflammation may play an important role. UA increases high mobility group box chromosomal protein 1 (HMGB1) expression and extracellular release in endothelial cells. HMGB1 is an inflammatory cytokine that interacts with the receptor for advanced glycation end products (RAGE), inducing an oxidative stress and inflammatory response, which leads to endothelial dysfunction. In this study, human umbilical vein endothelial cells (HUVECs) were incubated with a high concentration of UA (20 mg/dL) after which endothelial function and the expression of HMGB1, RAGE, nuclear factor kappa B (NF-*κ*B), inflammatory cytokines, and adhesion molecules were evaluated. UA inhibited endothelial nitric oxide synthase (eNOS) expression and nitric oxide (NO) production in HUVECs, increased intracellular HMGB1 expression and extracellular HMGB1 secretion, and upregulated RAGE expression. UA also activated NF-*κ*B and increased the level of inflammatory cytokines. Blocking RAGE significantly suppressed the upregulation of RAGE and HMGB1 and prevented the increase in DNA binding activity of NF-*κ*B and the levels of inflammatory cytokines. It also blocked the decrease in eNOS expression and NO production induced by UA. Our results suggest that high concentrations of UA cause endothelial dysfunction* via* the HMGB1/RAGE signaling pathway.

## 1. Introduction

Uric acid (UA), a final breakdown product of purine metabolism, is degraded by urate oxidase to allantoin and freely excreted in the urine in most mammals [1]. However, the gene for urease in humans is a nonfunctioning pseudogene, and overproduction or decreased excretion of UA results in uniquely high level of serum UA and sometimes hyperuricemia [2–4]. Hyperuricemia not only is intimately associated with gout but also has a close connection with many other diseases, especially with cardiovascular disease [5–7]. An elevated serum level of UA in humans is associated with systemic inflammation [8], endothelial dysfunction [9], hypertension [10], and cardiovascular disease [11]. Many studies have demonstrated that hyperuricemia is an independent risk factor for cardiovascular disease [12, 13]. It is well known that hyperuricemia is one of the main risk factors for endothelial dysfunction [14, 15], in which oxidative stress and inflammation may play an important role [16–18].

The receptor for advanced glycation end products (RAGE), a transmembrane multiligand receptor of the immunoglobulin superfamily, has been implicated in many chronic diseases [19, 20], including atherosclerosis, which is also believed to be an inflammatory disorder [21]. RAGE has been linked with atherosclerosis due to its expression on the surface of a wide variety of cells, such as endothelial cells, lymphocytes, monocyte-derived macrophages, and vascular smooth muscle cells, which are implicated in the pathogenesis of atherosclerosis [22]. In addition, blockade of RAGE signaling had significantly reduced progression of atherosclerosis, and the accumulation of RAGE-ligands was also reduced [23]. The interaction of RAGE and its diverse ligands, such as advanced glycation end products (AGEs), some S100s, amyloid peptide, and high mobility group box chromosomal protein 1 (HMGB1), stimulates oxidative stress generation and leads to cellular dysfunction [24]. There is growing evidence to suggest that the RAGE-ligands axis play an important role in the pathogenesis of cardiovascular disease [25, 26].

As a high affinity ligand of RAGE, HMGB1 is a recently discovered important extracellular mediator in systemic inflammation [27]. HMGB1 is secreted as a late mediator, with a delayed release during inflammation relative to classical early cytokines like tumor necrosis factor- (TNF-) *α*. HMGB1 participates in the pathogenesis of systemic inflammation after the early mediator response has resolved [28]. HMGB1 can be released by endothelial cells and mediates proinflammatory responses of endothelial cells [29]. High concentrations of UA significantly increased mRNA expression and extracellular release of* HMGB1* from human umbilical vein endothelial cells (HUVECs) [30]. Extracellular HMGB1 binding to RAGE activates nuclear factor kappa B (NF-*κ*B) which leads to proinflammation. In this study, we investigated whether the HMGB1/RAGE signaling pathway contributes to endothelial dysfunction induced by UA.

## 2. Materials and Methods

### 2.1. Cell Culture

HUVECs were obtained from China Center for Type Culture Collection. Uric acid crystallites (Sigma Life Science, St. Louis, MO, USA) were dissolved in 1 N NaOH solution. HUVECs were cultured on gelatin-coated 25 cm^2^ culture bottles and propagated in Dulbecco's modified eagle medium with 1.0 g/L glucose supplemented with 10% heat-inactivated fetal bovine serum. HUVECs were incubated at 37°C in a humidified atmosphere of 95% air and 5% carbon dioxide. The medium was refreshed every 2 to 3 days.

### 2.2. UA Treatment

HUVECs were cultured with 20 mg/dL UA solution for different times (0, 12, 24, and 48 h; the zero time means the point of first incubation with UA). Inhibition of the interaction between HMGB1 and RAGE was evaluated by using a specific RAGE blocking antibody (1 : 500 v/v, R&D Systems, Minneapolis, USA). HUVECs were cultured with anti-RAGE antibody (5 *μ*g/mL) for 2 h before UA (20 mg/dL) stimulation. For controls, HUVECs were cultured with control medium, a normal goat IgG control (5 *μ*g/mL), or medium with a control antibody (5 *μ*g/mL) and UA (20 mg/dL).

### 2.3. RNA Extraction and Fluorogenic Quantitative Polymerase Chain Reaction

Total RNA was extracted from HUVECs using the TRIzol reagent (Invitrogen, Carlsbad, CA, USA) according to the manufacturer's instructions. The RNA integrity was checked by nucleic acid agarose gel electrophoresis, and the RNA concentration was determined by ultraviolet spectrophotometer. Briefly, 1 *μ*g of total RNA for each sample was used to synthesize cDNA with an EasyScript® First-Strand cDNA Synthesis SuperMix (TransGen Biotech, Beijing, China) according to the manufacturer's protocol. Fluorogenic quantitative PCR (FQ-PCR) was performed using the StepOne™ Real-Time PCR System (Thermo Fisher Scientific, Rockford, IL). Primers were designed and synthesized by Shanghai Sangon Biotech (Shanghai, China). There were five pairs of primers. The primer sequences for* HMGB1* were 5′-GGGATGGCAAAGTTTTTCCCTTTA-3′ and 5′-CACTAACCCTGCTGTTCGCT-3′. For* RAGE*, the primer sequences were 5′-GCTTGGAAGGTCCTGTCTCC-3′ and 5′-CACGGACTCGGTAGTTGGAC-3′. For intercellular adhesion molecule-* (ICAM-) 1*, the primer sequences were 5′-CACAGTCACCTATGGCAACG-3′ and 5′-GTGTCTCCTGGCTCTGGTTC-3′. For vascular adhesion molecule-* (VCAM-) 1*, the primer sequences were 5′-AGTTGAAGGATGCGGGAGTA-3′ and 5′-GTGTCTCCTGGCTCTGGTTC-3′. The mRNA expression of glyceraldehyde 3-phosphate dehydrogenase (*GAPDH*) was used as an internal reference.

### 2.4. Western Blotting Assay

RAGE, endothelial nitric oxide synthase (eNOS), ICAM-1, and VCAM-1 were examined in cell samples by western blotting. The extracellular HMGB1 was concentrated using cellulose 3,000 molecular weight cut-off (MWCO) concentrating centrifugal filter units (Millipore Corp, Billerica, MA, USA) prior to western blotting. NF-*κ*B was examined in nuclear and plasma protein samples by western blotting. Nuclear protein and cytoplasm protein fractions were obtained using a nuclear plasma protein extraction kit (CWBio, Beijing, China) following the manufacturer's instructions. The protein samples were dissolved in Laemmli buffer, boiled for 10 min at 100°C, and equal amount of proteins was separated on 10% SDS-PAGE gels (Bio-Rad Laboratories, Inc. USA). The membrane was blocked in 5% wt/vol skim milk for 1 hour after the membrane was transferred to a nitrocellulose membrane (Cell Scientific). The proteins were incubated with primary antibodies and followed with secondary antibodies. Polyclonal rabbit antibodies against HMGB1 (Abcam Inc., Cambridge, MA, USA), RAGE (Abcam Inc.), NF-*κ*B p65 (Santa Cruz Biotechnology, Santa Cruz, CA), eNOS (Abcam Inc.), ICAM-1 (Affbiotech., Cincinnati, OH, USA), VCAM-1 (BOSTER, Wuhan, Hubei, China), proliferating cell nuclear antigen (PCNA; Proteintech, Wuhan, Hubei, China), and a monoclonal mouse antibody to *β*-actin (Affbiotech.) were used as primary antibodies according to the manufacturers' protocol (including dilutions). Horseradish peroxidase-conjugated anti-rabbit and anti-mouse secondary antibodies (ZSGB-BIO, Beijing, China) were used. The proteins were detected using enhanced chemiluminescence reagents (Thermo Fisher, Scientific, Rockford, IL). The images were captured through Gel Doc XR system (Bio-Rad, Hercules, CA, USA) and analyzed using Image Lab software (vision 4.0). Anti-*β*-actin antibody was used as the loading control for total proteins and cytoplasm protein, and the relative protein levels of nuclear protein were calculated as densitometric ratios to PCNA.

### 2.5. Measurement of Nitric Oxide Release

Nitric oxide (NO) levels in cell culture supernatants were measured with Griess reagent by the nitrate reductase method using a nitric oxide assay kit (Nanjing Jiancheng, Nanjing, China) according to the manufacturer's protocol.

### 2.6. Measurement of Cytokines by Enzyme-Linked Immunosorbent Assay (ELISA)

Immunoreactive TNF-*α* and IL-6 were measured in duplicate using ELISA kits according to the manufacturer's instructions (ExcellBio, Shanghai, China).

### 2.7. Statistical Analysis

Data are expressed as means ± standard deviation (SD). Differences among groups were analyzed by two-tailed Student's *t*-test or one-way ANOVA followed by post hoc Dunnett's test, as appropriate. All statistical analyses were two-sided, and a *P* value of less than 0.05 was considered statistically significant. Statistical analyses were carried out using SPSS version 17 (SPSS Inc., Chicago, IL, USA).

## 3. Results

### 3.1. A High Concentration of UA-Induced Endothelial Dysfunction

**Figure 1:**
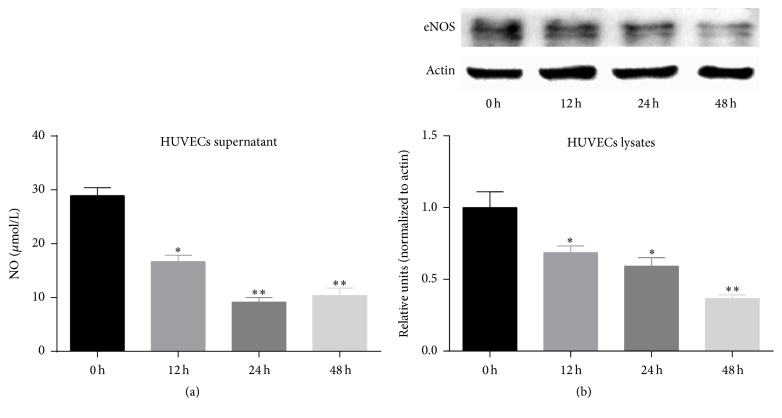
A high concentration of UA (20 mg/dL) induces endothelial dysfunction. (a) UA significantly reduced NO release from HUVECs in a time-dependent manner. (b) UA significantly reduced eNOS protein expression of HUVECs in a time-dependent manner. Data are expressed as means ± SD, ^*∗*^ *P* < 0.05, ^*∗∗*^ *P* < 0.01 versus 0 h group.

To investigate whether a high concentration of UA could induce endothelial dysfunction, we detected the changes in the amount of NO release and the expression eNOS protein in HUVECs treated with 20 mg/dL UA for different time periods. When HUVECs were stimulated with UA for 24 h, the amount of NO release was significantly reduced versus control cells (*P* < 0.05) (Figure 1(a)), as was the expression of eNOS protein (*P* < 0.05) (Figure 1(b)). These results show that a high concentration of UA can reduce the expression level of eNOS and the amount of NO released by HUVECs, which leads to endothelial dysfunction.

### 3.2. A High Concentration of UA Upregulates the Expression of RAGE and HMGB1 in HUVECs, Accompanied by an Increase in Released HMGB1

**Figure 2:**
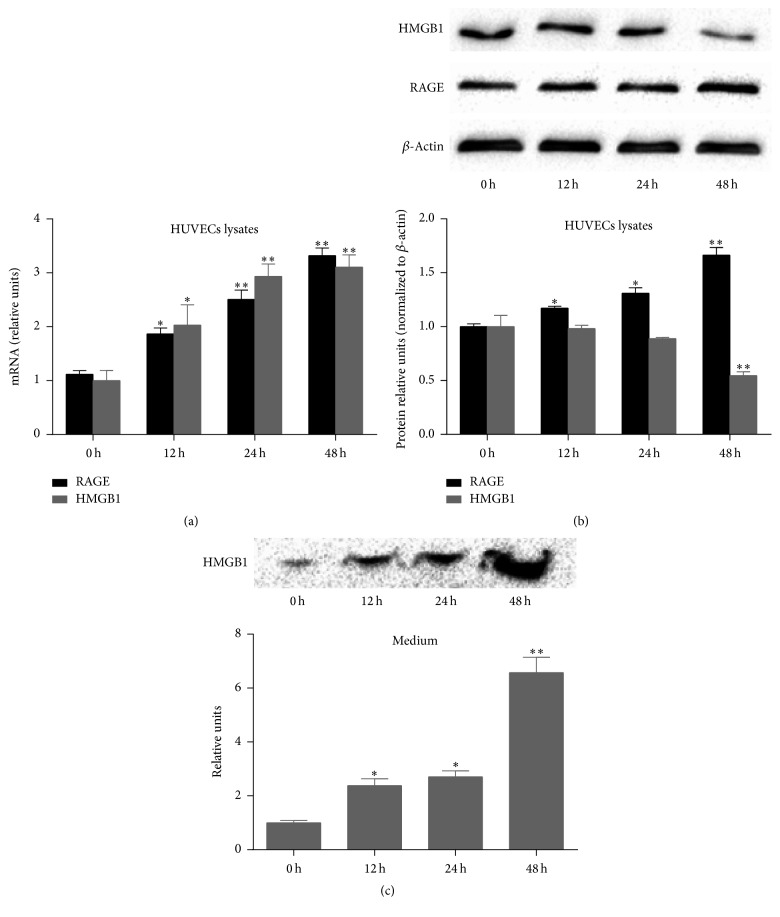
A high concentration of UA (20 mg/dL) upregulates the expression of RAGE and HMGB1 in HUVECs, accompanied by an increase in released HMGB1. (a) UA significantly upregulated the mRNA expression RAGE and HMGB1 in a time-dependent manner. (b) UA significantly upregulated the protein expression of RAGE in a time-dependent manner, while the protein expression of HMGB1 decreased. (c) UA significantly increased release of HMGB1 protein into the extracellular fluid. Data are expressed as means ± SD, ^*∗*^ *P* < 0.05, ^*∗∗*^ *P* < 0.01 versus 0 h group.

To examine whether a high concentration of UA can upregulate the expression of RAGE and HMGB1, we detected the mRNA and protein expression of RAGE and HMGB1 by FQ-PCR and western blotting assay in HUVECs treated with 20 mg/dL UA. When the HUVECs were stimulated with 20 mg/dL UA, the mRNA expression of RAGE and HMGB1 significantly increased in a time-dependent manner (Figure 2(a)). At the same time, the protein expression of RAGE gradually increased, while the protein expression of HMGB1 decreased (Figure 2(b)). Therefore, in subsequent experiments, the extracellular level of HMGB1 was detected by western blotting assays. We found that UA increased the extracellular level of HMGB1 in a time-dependent manner (Figure 2(c)). The above results show that a high concentration of UA promotes the production and release of HMGB1 protein into the extracellular fluid.

### 3.3. A High Concentration of UA Activates NF-*κ*B and Inflammation Signals in HUVECs

**Figure 3:**
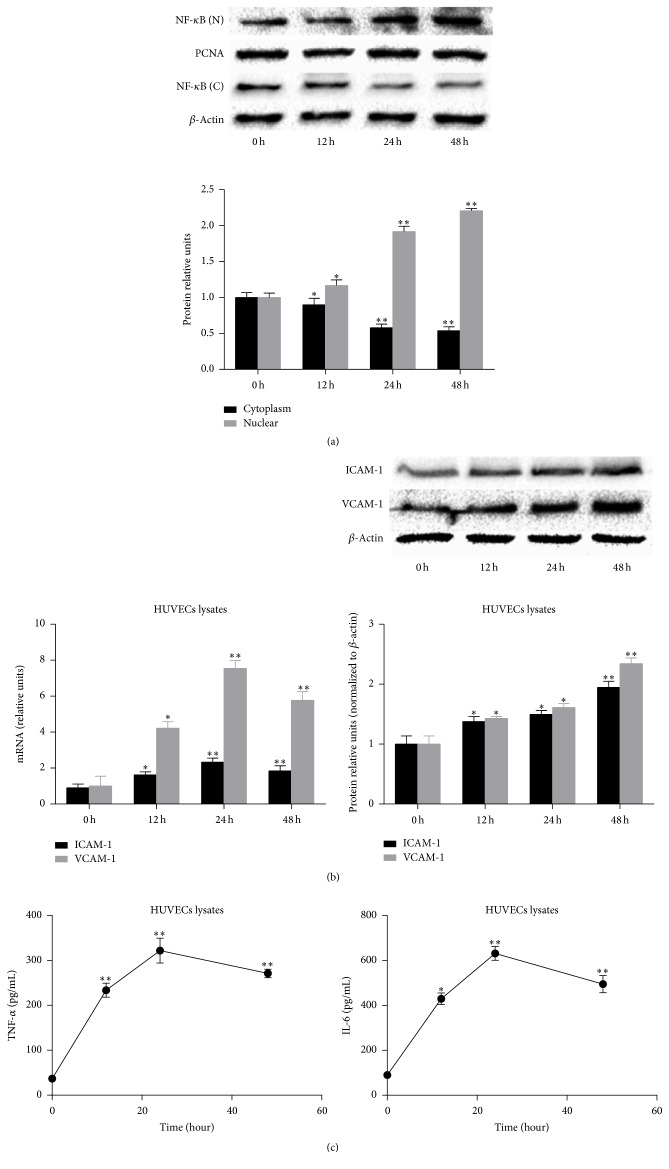
A high concentration of UA (20 mg/dL) activates NF-*κ*B and inflammation signals in HUVECs. (a) UA significantly decreased the expression of NF-*κ*B in the cytoplasm of HUVECs and significantly increased the expression of NF-*κ*B in the nucleus of HUVECs in a time-dependent manner. (b) UA significantly increased the mRNA and protein expression of ICAM-1 and VCAM-1 in HUVECs in a time-dependent manner. (c) UA significantly increased the levels of TNF-*α* and IL-6 release from HUVECs. Data are expressed as means ± SD, ^*∗*^ *P* < 0.05, ^*∗∗*^ *P* < 0.01 versus 0 h group.

To clarify whether RAGE/HMGB1 interactions can activate NF-*κ*B, the expression of NF-*κ*B p65 in the cytoplasm and nucleus of HUVECs was detected by western blotting assays. After treatment with 20 mg/dL UA, we observed a decrease in cytoplasmic NF-*κ*B (Figure 3(a)) in a time-dependent manner.

To examine whether UA induces inflammatory cytokines and adhesion molecules, which participate in the pathogenesis of atherosclerosis, we detected the expression of ICAM-1 and VCAM-1 in HUVECs assessed by FQ-PCR and western blotting assays and the TNF-*α* and IL-6 release from HUVECs measured by ELISA. After treatment with 20 mg/dL UA, the mRNA and protein expressions of ICAM-1 and VCAM-1 in HUVECs significantly increased in a time-dependent manner (Figure 3(b)), and the TNF-*α* and IL-6 release from HUVECs also significantly increased in a time-dependent manner (Figure 3(c)). The above results show that a high concentration of UA activates NF-*κ*B and inflammation signals in endothelial cells, which has an important role in the pathogenesis of atherosclerosis.

### 3.4. Blockage of RAGE Suppresses Endothelial Dysfunction and the HMGB1/RAGE Signaling Pathway Induced by a High Concentration of UA

**Figure 4:**
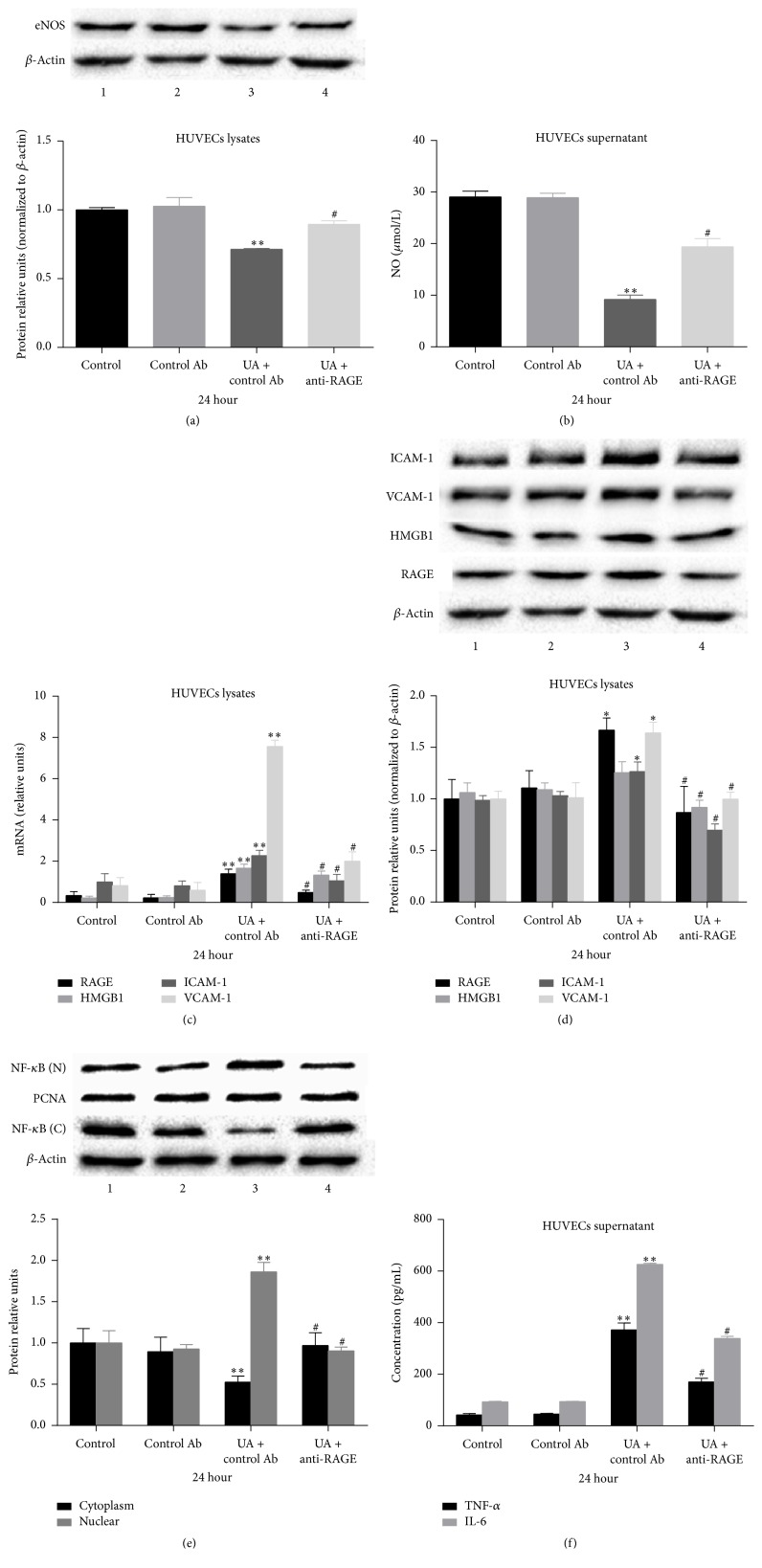
Blockage of RAGE suppresses endothelial dysfunction and the HMGB1/RAGE signaling pathway induced by a high concentration of UA. ((a) and (b)) Treatment of HUVECs with anti-RAGE antibody for 24 h significantly blocked the decrease in eNOS expression and NO production induced by 20 mg/dL UA. ((c) and (d)) Treatment of HUVECs with anti-RAGE antibody for 24 h significantly suppressed the increase in the mRNA and protein expression of RAGE, HMGB1, ICAM-1, and VCAM-1 induced by 20 mg/dL UA. (e) Treatment of HUVECs with anti-RAGE antibody for 24 h significantly prevented the increase in DNA binding activity of NF-*κ*B. (f) Treatment of HUVECs with anti-RAGE antibody for 24 h significantly decreased the release of IL-6 and TNF-*α* induced by 20 mg/dL UA. Data are expressed as means ± SD, ^*∗*^ *P* < 0.05, ^*∗∗*^ *P* < 0.01 versus the untreated control, and ^#^ *P* < 0.05 versus the UA + control Ab.

To investigate the role of RAGE in endothelial dysfunction induced by UA, we used a specific antibody targeted against RAGE (anti-RAGE antibody) to neutralize RAGE. Treatment of HUVECs with anti-RAGE antibody for 24 h significantly blocked the decrease in eNOS expression and NO production induced by 20 mg/dL UA (Figures 4(a) and 4(b)). These results show that endothelial dysfunction induced by UA is partly mediated via RAGE.

To further determine whether the expression of HMGB1 and the activation of NF-*κ*B and inflammation signals in endothelial cells induced by UA were mediated by RAGE, we evaluated the effects of anti-RAGE antibody on the expression of RAGE, HMGB1, NF-*κ*B, ICAM-1, and VCAM-1 and the release IL-6 and TNF-*α* from HUVECs. Treatment of HUVECs with anti-RAGE antibody for 24 h significantly suppressed the upregulation of RAGE, HMGB1, ICAM-1, and VCAM-1 (Figures 4(c) and 4(d)), prevented the increase in DNA binding activity of NF-*κ*B (Figure 4(e)), and decreased the release of IL-6 and TNF-*α* induced by 20 mg/dL UA (Figure 4(f)). The above results show that the proinflammatory activity of a high concentration of UA is partly mediated via RAGE in endothelial cells.

## 4. Discussion

In this study, we demonstrated a novel mechanism of high concentration UA-induced endothelial dysfunction. UA inhibited eNOS expression and NO release in endothelial cells and increased the levels of inflammatory cytokines by stimulating the HMGB1/RAGE signaling pathway. To our knowledge, this is the first time a link between UA-induced endothelial dysfunction and the HMGB1/RAGE signaling pathway has been established.

Hyperuricemia not only is the etiological factor of gout [17, 31], but also can lead to many different diseases [32]. Many studies have demonstrated that hyperuricemia has a close relationship with cardiovascular diseases and is an independent risk factor for cardiovascular diseases [12, 13, 33, 34]. It has been supposed that endothelial dysfunction plays an important role in the initiation of atherosclerosis [35] and is an early marker for atherosclerosis [36]. Many studies have shown that oxidative stress and inflammatory responses are the main pathogenesis of UA-induced vascular endothelial dysfunction [37–39]. Hyperuricemia, through inducing oxidative stress and inflammation, reduces the expression of eNOS and NO synthesis, leading to damage of endothelial function [9]. In the present study, we also found that a high concentration of UA downregulated the expression of eNOS and decreased the production of NO by HUVECs and also increased the levels of inflammatory cytokines (IL-6 and TNF-*α*) and adhesion molecules (ICAM-1 and VCAM-1). These results demonstrate that high concentrations of UA can induce inflammatory responses and result in endothelial dysfunction. However, the exact mechanisms have not been described yet.

**Figure 5:**
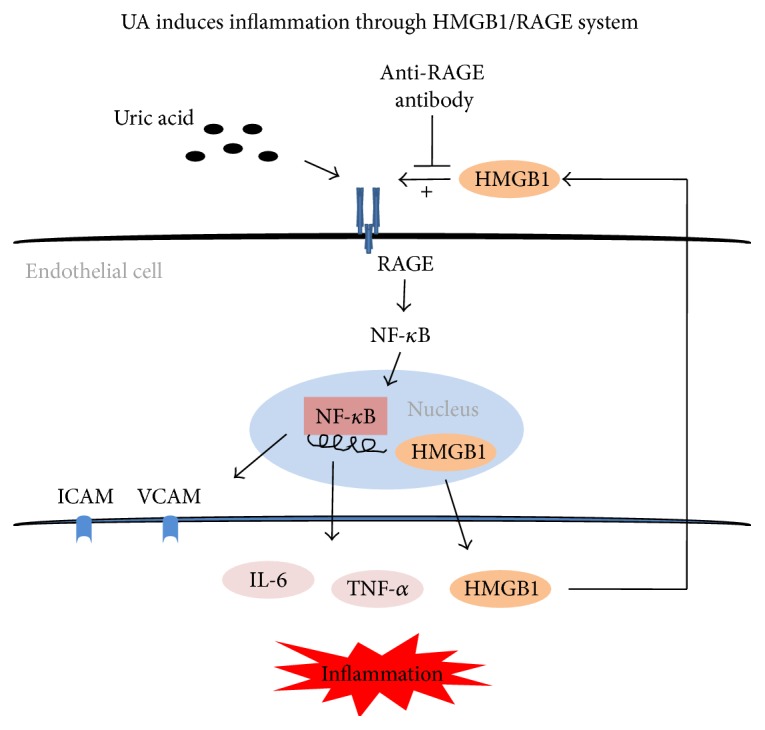
Potential mechanism of UA-induced inflammation in endothelial cell. High uric acid stimulates the RAGE signaling pathway and activates NF-*κ*B, which results in the production and release of proinflammatory cytokines, including the expression and extracellular release of HMGB1 in endothelial cells. As a high affinity ligand of RAGE, HMGB1 interacts with RAGE and contributes to the amplification of the inflammatory response and finally induces endothelial dysfunction. Blockade of RAGE by anti-RAGE antibody can suppress the HMGB1/RAGE signaling pathway, therefore alleviating endothelial dysfunction.

There is a large body of evidence to suggest that the RAGE-ligands axis has an important role in the pathogenesis of atherosclerosis. RAGE is a multiligand receptor of the immunoglobulin superfamily, which has been implicated in many chronic inflammation diseases, including atherosclerosis [40]. Previous people have found that diabetic apoE^−/−^ mice showed increased plaque area as well as increased expression of RAGE and its ligands, but diabetic RAGE^−/−^/apoE^−/−^ mice showed significantly reduced plaque accumulation and expression of RAGE and its ligands [23]. The studies in vivo and in vitro have demonstrated that the interaction between RAGE and its ligands induces inflammation and causes many chronic diseases, including atherosclerosis [41, 42]. As a high affinity ligand of RAGE, HMGB1 belongs to the group of endogenous damage-associated molecular pattern molecules, which are often associated with sterile inflammation [43]. Extracellular HMGB1 participates in various chronic diseases, and HMGB1/RAGE signaling pathway is one of the major signaling pathways about these diseases [44]. RAGE activation by extracellular HMGB1 leads to nuclear translocation of NF-*κ*B in HUVECs and further promotes the production and release of proinflammatory mediates and contributes to the amplification of the inflammatory response and finally induces endothelial dysfunction [45]. Treatment of HUVECs with a high concentration of UA results in increased mRNA expression and extracellular release of HMGB1 [30], which induces proinflammatory responses and endothelial dysfunction [29]. Interactions between RAGE and HMGB1 stimulate oxidative stress and inflammatory response, leading to endothelial dysfunction [46, 47]. In the present study, we found that a high concentration of UA increased intracellular HMGB1 expression and extracellular secretion while also increasing RAGE expression and activating NF-*κ*B signaling in endothelial cells. We also found that blocking (by anti-RAGE antibody) RAGE significantly suppressed the upregulation of RAGE and HMGB1 and inhibited the increase in DNA binding activity of NF-*κ*B and the levels of inflammatory cytokines (IL-6 and TNF-*α*) and adhesion molecules (ICAM-1 and VCAM-1) induced by a high concentration of UA. At the same time, blockage of RAGE significantly prevented the decrease in the eNOS expression and the NO production induced by a high concentration of UA. These results imply that UA induces endothelial dysfunction by stimulating the HMGB1/RAGE signaling pathway (Figure 5).

## 5. Conclusions

In conclusion, the present study indicated that high concentrations of UA induce endothelial dysfunction by the HMGB1/RAGE signaling pathway. These results provide new insight into the mechanisms of UA-induced endothelial dysfunction and atherosclerosis.
